# Prevalence and barriers to male involvement in antenatal care in Dar es Salaam, Tanzania: A facility-based mixed-methods study

**DOI:** 10.1371/journal.pone.0273316

**Published:** 2022-08-19

**Authors:** Bosco Mapunda, Furaha August, Dorkas Mwakawanga, Isaya Mhando, Andrew Mgaya

**Affiliations:** 1 Department of Obstetrics and Gynaecology, Muhimbili National Hospital, Dar es Salaam, Tanzania; 2 Department of Obstetrics and Gynaecology, Muhimbili University of Health and Allied Science, Dar es Salaam, Tanzania; 3 Department of Nursing and Midwifery, Muhimbili University of Health and Allied Science, Dar es Salaam, Tanzania; 4 Department of Obstetrics and Gynaecology, St. Joseph College of Health and Allied Sciences, Dar es Salaam, Tanzania; 5 Department of Women’s and Children’s Health/International Maternal and Reproductive Health and Migration, Uppsala University, Uppsala, Sweden; Federal University of Mato Grosso do Sul, BRAZIL

## Abstract

**Background:**

Men have traditionally not been fully involved in reproductive health care of their partners, and yet, they play a crucial role in family decision-making and therefore crucial key players in preventing poor pregnancy outcomes. This study aimed to assess prevalence and determinants of male participation in maternal health care and explore male partners’ perspective of their involvement in antenatal care at an urban tertiary referral facility.

**Methods:**

A mixed-methods study was conducted from October 2018 to January 2019 at Muhimbili National Hospital. A cross-sectional survey of 428 nursing mothers and two focus group discussions of male partners (n = 7 and n = 11) of women attending antenatal clinic and nursing mothers in the post-natal ward were performed. Using SPSS Ver. 23 (IBM, Chicago, IL), frequency distribution tables summarized demographic data and categories of male partners’ involvement in antenatal care. Focus group discussions included male partners of age from 24 to 55 years at their first to fifth experience of pregnancy and childbirth. Interviews were audio-recorded, and then transcribed and coded. Thematic analysis was applied.

**Results:**

The prevalence of male involvement in antenatal care was 69%. More than two-thirds of nursing mothers received physical, psychological and financial support from partners (76%) and attended four or more antenatal visits (85%). Five themes of male perspective of their involvement in antenatal care were generated, including: a) cultural norms and gender roles, b) ignorance of reproductive health service, c) factors outside their control, d) couple interaction and conflicts, and e) institutional obstacles.

**Conclusion:**

The prevalence of male partners’ involvement in antenatal care was relatively high. Men’s involvement in antenatal care depended on access to antenatal care education, standards of structure and process of antenatal service and how well their role was defined in the maternal health care system. Interactions and practice in society, employment sector and government health system should complement strategies to promote men’s involvement in maternal health.

## Background

Regional disparities in quality of maternal health care include substandard reproductive health services in developing countries compared to those in developed countries [1]. Socioeconomic, cultural, religious and ethnic disparities continue to inhibit women’s ability to make decisions regarding their own health [2–4] because of men’s control of allocation of family income, transportation and time, and access to health services. Male involvement in maternal health services remains a challenge to safe motherhood [5], despite its essential roles in providing financial, emotional and physical support to women [6–8]. Efforts to engage male partners in maternity care not only prevent delays in receiving appropriate care but also facilitate adequate treatment at the appropriately-equipped health facility level.

In Tanzania, Reproductive and Child Health (RCH) services cut across a health-system pyramid where primary health care is at the base and specialized tertiary care at the apex. As a developing country at a low middle income level, the average male involvement in RCH services ranges from 60 to 70% [9, 10], despite a mandate under national RCH policies and guidelines for male participation [10]. Similar rates of male involvement in RCH services were evident in other African settings including Kenya [11], Ethiopia [12] and Ghana [13]. Maternal and child health care has been perceived as women’s rather than men’s affair [14–16]. Apart from the misconceived gender role, other inhibitions of male partners’ attendance to antenatal clinic included unfriendly structure of services [17–19], fear of testing for Human Immunodeficiency Virus (HIV) for self-suspected or confirmed HIV infection [9, 10, 20], and ill-perceived process of care, including waste of time and loss of earnings while waiting for service [7, 18, 19]. Engaging male partners in maternal health care require in-depth understanding of social cultural impact of each intervention. Further, persistent gender inequality especially in Sub-Saharan Africa, including Tanzania, where men are decision makers and gatekeepers for families [16, 21] necessitates a new approach that harmonizes incorporation of men in a culturally-perceived female domain. However, in some settings, interventions to encourage male partners unintentionally discriminated against unaccompanied women that may either be single or in a bad relationship [22]. Thus, interventions to solicit male involvement in antenatal care require adequate buy-in from both couples and health care providers, and continuous updating of context-specific cultural influences.

Determination of barriers of male participation in maternal health care is an important step in meeting men’s needs for supporting women’s health, and subsequently improving family health. As a quality-improvement measure, interventions to improve male participation in maternal health requires continuous inquiry and feedback using locally-defined quality indicators and qualitative interviews that provide men’s and women’s experience in each setting. Therefore, a deeper understanding of needs of male partners and objective assessment of complex phenomena that affect their participation in maternal health service [23] in a measurable frame work [24] is a prerequisite. This study aimed to assess prevalence and determinants of male participation in antenatal care and explore male partners’ perspective of their involvement in antenatal care at an urban tertiary referral facility. It also aims to inform the health system of detailed, context-specific stakeholders’ perspectives and desired level of standards of implemented care, in order to improve male involvement in antenatal care using demand-driven interventions and a measurable evaluation framework.

## Material and methods

### Study design and settings

A mixed-methods study was conducted at Muhimbili National Hospital (MNH) [25] using a cross-sectional survey of nursing mothers during the postnatal period of the first 24 hours after childbirth, from October 2018 to January 2019. During the period of the study, qualitative exploration of opinion of male partners was also performed using two focus group discussions (FGD), one of male partners of nursing mothers admitted in post-natal ward and another of male partners of women attending antenatal clinics at MNH. MNH is a tertiary referral health facility serving the city of Dar es Salaam and neighboring regions including Lindi, Mtwara, Pwani, Zanzibar and Morogoro. As a teaching university hospital, MNH also serves as a medical training facility for numerous universities in Tanzania [25]. Like other public health facilities, the cost of RCH service at MNH has user-fee exemption and cost sharing modalities [26, 27] for clients who were referred from public-referral hospitals. Self-referred clients are received as private clients (either health-insured or paying services in cash) under Intramural Private Practice Management (IPPM). Approximately 9,000 deliveries are conducted per year for both public (60%) and private (40%) clients at a caesarean section rate of 54%.

RCH services are provided in two separate maternity buildings—Maternity ‘One’ and ‘Two’, which are in close proximity. The ground floor of the Maternity Two building accommodates outpatient RCH services including a registration counter and a waiting hall for 60 to 80 people, eight antenatal and postnatal clinic consultation rooms, two privately-secured rooms for HIV/AIDS counselling and testing, two rooms for family planning counselling and provision of contraceptives, a room equipped for minor procedures and a laboratory for routine test (such as haemoglobin, urine analysis, random blood glucose and rapid testing for malaria, syphilis and hepatitis B surface antigen). Outpatient RCH services are provided five times a week from Monday to Friday, from 9 a.m. to 5 p.m. Public outpatients are registered from 9 a.m. while private outpatients start the registration process at 12 p.m. RCH services for public and private clients are comparable, except that private clients have a privilege of choosing specialist service and receiving more comfortable in-patient accommodation in dedicated private wards. Other floors of Maternity Two constitute inpatient wards for antenatal and postnatal mothers under IPPM and nursing mothers performing Kangaroo Mother Care. Maternity One is mostly for antenatal and postnatal inpatients care for cost-sharing and user fee -excepted clients. Other functions in Maternity One include sonographic imaging services, pharmacy services, cashiers’ counter and inpatient registration counter for both maternity buildings.

Tanzania is a low-middle income country divided into 30 regions, among which Dar es Salaam is the largest business city with a population of over 6 million people living in four municipalities (Kinondoni, Ubungo, Temeke and Kigamboni) and one city–Ilala City, where MNH is located. The health system pyramid puts MNH at the apex of an inclusive network of mainly government-owned health facilities organized in such a way that dispensaries and health centers serve most of the population as primary health care facilities, while district, regional and specialized/consultant hospitals serve as referral health facilities. MNH is the highest referral health facility in the eastern and coastal zone health system, as well as supervising and mentoring other referral health facilities in western and northern lake zone, northern zone and southern zone. All levels of public and some private health facilities provide RCH services, including antenatal and postnatal care, under-five care, family planning counselling and provision of contraception, essential vaccinations services, HIV/AIDS counselling and testing, and Emergency Obstetric and Newborn Care (EmONC).

### Study population, sampling, recruitment and data collection

#### Quantitative methods

All nursing mothers in the postnatal wards were assessed for eligibility for recruitment including antenatal care attendance at MNH. Women who delivered within 24 hours, those who were either too sick to be interviewed or those who refused to participate in the study, were excluded. Using Kish Leslie’s formula, n = (z^2 p(100-p))/ε^2 by assuming that the proportion of male involvement in maternal healthcare in Tanzania (p) was 40% [28] at a maximum error (ε) of 5%, z value at 95% level of confidence of 1.96 and 10% non-response rate, the minimum required sample size was 413.

The first author and research assistants (trained nurses) identified women from postnatal ward using inpatient registers and ward round records. Inclusion criteria was applied by assessing the antenatal cards. Eligible participants were listed and gave written consent. A simple random sampling technique (lottery) was performed by asking eligible participants to randomly choose a piece of paper from an envelope (A4 size) filled with similarly folded and mixed-up pieces of paper, according to number of eligible participants in a particular ward. Each piece of paper was either written “YES” (70%) or ‘NO” (30%). Participants who picked papers that were written “YES” were included in the study and subsequently filled out the questionnaires. those who picked papers written “NO” were excluded from the study. The same procedure was instituted in all postnatal wards until the sample size of 428 participants was reached.

Data collection was conducted using a structured questionnaire adapted from previous study [28]. The questionnaire covered these topics: a) Demographic parameters such as age, marital status, religion, level of education, occupation, duration of relationship (physical, social or financial) and number of children; b) information on barriers of male involvement in ANC of which women were to choose one or more possible barriers [11, 12, 29] such as ‘being too busy with work’, ‘thinking its women’s’ affairs’, ‘lack of maternal health knowledge’ and health service related factors such as ‘comfortability to be in antenatal clinic’, ‘prohibitions to participate during care’, ‘poorly perceived quality of care’, ‘staff attitudes’, ‘inhibitive facility infrastructure’ and /or, ‘long waiting time’. Questionnaires were screened for completeness and missing information was inquired before data entry, which was concomitantly performed with data collection.

#### Qualitative methods

The study conducted a purposeful sampling of male partners of women attending postnatal clinics and others who were nursing mothers in postnatal wards. The male partners were simultaneously selected during a cross-section survey, and their selection was independent of their female partners’ participation in the study. Eligible participants who agreed to be interviewed signed a written consent for participation. Two FGDs were performed. The first FGD was of male partners of nursing mothers in the postnatal ward (n = 7). Male participants were between 24 to 52 years of age, and they had experienced one to four pregnancies with their spouses. Three out of seven participants were self-employed, two were employed in private organizations, and the rest were government employees. The second FGD was of male partners of women attending postnatal clinic (n = 11). Participants’ ages were between 30 and 55 years and they had experienced one to five pregnancies with their spouses. Seven out of eleven participants were self-employed, while the rest were employed in private organizations.

Each FGD was conducted in a closed–door session for about 1 hour. The first author was a passive member of the group. All FGDs were audio recorded and transcribed verbatim by the first author on the same day. The transcriptions were in Kiswahili and later translated to English.by the first author with and later verified by other researchers. Participants used Kiswahili during the FGDs. All researchers were Kiswahili speakers, as the first language, which is also the language normally spoken by clients and service providers in the hospital. The questions posed during the FGDs were open-ended and focused on these topics: a) general understanding of being a father and biological father, b) benefits of male partner’s involvement, c) barriers of male partner’s involvement, d) whether health service related factors such as facility infrastructure, long waiting time, staff attitudes and quality of care affect their antenatal clinic attendance, and e) recommendations on how to tackle those barriers. Follow-up questions were put forward regarding staff interaction with male partners and quality of customer care. The first FGD transcript was read and areas that needed in depth inquiry and further exploration were identified and introduced in the second FGD. Saturation was reached at a point where no new information was gathered from the FGDs. The participants were given soft drinks during the discussions as a gesture of appreciation for participating [30].

### Data analysis

Quantitative data was entered into SPSS ver. 23 (IBM SPSS, Chicago, IL). Data entry and cleaning included amending information that was incomplete or suspected of being incorrect by cross-checking with women before discharge. Typographic errors and duplicated information were removed. Simple descriptive statistical analysis was used to obtain percentage distribution of main outcome measure associated with male involvement in ANC. Male involvement was defined using a composite scoring system based on five factors that were weighted a score of ‘1’ when present or ‘0’, when not. These factors included a) Did your husband attend antenatal clinic at least once? b) Was he available during scheduled days and times of antenatal care appointments? c) Did he participate in decision-making regarding pregnancy and childbirth? d) Did he provide emotional, physical or financial support during antenatal clinic? and e) Did he take part in any maternal healthcare education programs? Demographic, socioeconomic and maternal factors were tested for association with male involvement in antenatal care using chi-squire and bivariate logistic regression. Odds ratio with 95% confidence intervals of p < 0.05 was considered as significant for association.

For qualitative data, transcripts from the FGDs were translated in English prior to analysis to enable report-writing and dissemination for non-Kiswahili-speaking audience and readers. Thematic content analysis [31] was used to describe the themes within the FGD findings in stepwise manner by familiarizing with data, generating initial codes, searching for themes, reviewing themes, defining and naming themes and producing a report. Naturalistic inquiry guided the emergent analysis [32] from the initial data collection, where information from one FGD was used to generate new questions in the other. Topical saturation of new concepts was met upon repetition of participants’ answers. Qualitative data analysis Nvivo 10 computer software was used in managing and organizing data. Triangulation was performed to compare and contrast reported findings.

### Ethics approval and consent to participate

Ethical approval was obtained from Research and Publication Committee of Muhimbili University of Health and Allied Sciences (MUHAS) Senate (Ref. No. DA.287/298/01A). Approval to conduct the study at MNH was granted by MNH Research Ethics Review Board. All methods were performed in accordance with the relevant guidelines and regulations under MNH research policy. A written informed consent was obtained from all participants in Kiswahili. Participants were informed of their right to withdraw from the study at any point and that the information obtained was confidential. All recordings and transcripts were made anonymous before being discussed within the research group. Patients’ names or hospital registration numbers were not used to ensure confidentiality, and access to participants’ information was given to researchers only.

## Results

The majority of women (39%) in cross sectional survey (N = 428) were between 25–29 years of age (Table 1). A larger proportion of them were educated to college/university level (65%), and employed (63%) either by the government (34%) or private institutions (29%), compared to other subgroups in the categories. Almost all women were married or in a relationship for more than 1 year (90%). During the recent pregnancy and childbirth, most women reported to have received adequate physical, psychological and financial support from partners (76%) and attended four or more antenatal visits (85%). However, only 23% of all women attended at least four antenatal visits with their partners.

**Table 1:** Percentage distribution of background characteristics of studied group (N = 428).

Characteristics	Number (n)	Percentage (%)
*Age (yrs*.*)*		
Less than 20	33	7.7
20–24	0	0
25–29	165	38.5
30–34	145	33.9
35–39	65	15.2
Equal or more than 40	20	4.6
*Education level*		
No formal education	5	1.2
Primary school education	25	5.8
Secondary school education	124	29.0
College/University	274	65.0
*Occupation*		
Housewife	34	7.9
Government employee	123	28.7
Private institution employee	144	33.6
Self employed	127	23.7
*Marital status*		
Married/Cohabiting	387	90.4
Single	41	9.6
*Duration in relationship*		
Less than 1 year	43	10
1–5 years	249	58.2
6–10 years	98	22.9
More than 10 years	38	8.9
*Attendance of antenatal visits*		
Once	6	1.4
Twice	26	6.1
Three times	29	6.8
Four or more times	367	85.7

Sixty-nine per cent of women reported significant partner involvement in antenatal care (Table 2). Most of them attended at least one antenatal visit with their partner (72%). Similarly, majority of women reported to have had adequate access to their partners, when needed (90%), and received physical, psychological or financial support (76%). Almost all women reported male partner participation in joint decision-making (90%) and attended maternal health programs (75%) with their partners, during antenatal care.

**Table 2:** Percentage distribution of factors for male involvement in antenatal care in the studied group (N = 428).

Partners’ involvement	Number (n)	Percentage (%)
*Fulfilled significant involvement*		
Yes	294	68.7
No	144	31.3
*Fulfilled factors*		
Has your male partner ever attended ANC clinic with you?		
Yes	307	71.5
No	121	28.5
Was your partner accessible e.g. through physical address, phone, email etc?		
Yes	384	89.7
No	44	10.3
Was your partner able to provide adequate emotional, physical and financial support?		
Yes	234	75.9
No	103	24.1
Could your partner allow joint decision-making on maternal health?		
Yes	384	89.7
No	44	10.3
Was he able to take part in maternal healthcare programs		
Yes	322	75.2
No	106	24.8

Most women reported that lack of knowledge in maternal health care was the main reason for lack of male involvement (41%). Other reasons were male partner’s perception of maternal health as women’s affairs (35%), and being too busy to attend antenatal clinic visits (17%). A few women (4%) also reported facility related barriers including uncomfortable environment in antenatal clinic, prohibitive participation during care, poorly perceived quality of care, poor staff attitudes, inhibitive facility infrastructure and long waiting time, as contributors of lack of male involvement in antenatal care. None of the factors was independently associated with fulfillment of criteria of male involvement in antenatal care.

Five themes emerged from FGD with male partners with regards to barriers of their involvement in antenatal care, including these: Cultural norms and gender roles; ignorance of maternal health care; factor out of their control; couple interaction and conflicts; and institutional obstacles. The themes are described with quotes as follows:

### Cultural norms and gender roles

Male partners agreed that their involvement during antenatal care and childbirth is essential. They expressed a need for change from ‘African ways’ to accepting responsibility for supporting their spouses from early pregnancy to childbirth. Health education and doctors’ advice were motivational to antenatal clinic attendance. However, societal pressures were generally perceived as inhibitive to male involvement. As these participants said:

“*Therefore*, *our African societies are letting us down for thinking that reproductive health is for women*, *and it is not very necessary for men to get involved*”

(RA, 40-year-old, FG1)

Another participant also said:

“*I think*, *first of all*, *you have to prepare yourself after discovering that you are expecting a child*, *you should start going to the clinic*, *so that you get advice from doctors*”

(RM, 45-years-old, FG2)

Male involvement was considered an expression of love and care that echoed commitment to physical and emotional support for their pregnant partners. Participants expressed the importance of being a supportive partner. One said:

“*The benefit comes when your wife gives birth safely*. *She will be narrating to the child how you*, *the father*, *took good care of her during pregnancy including taking her to the clinic and physical exercises*. *This increase love in marriage and of the child to the father*”

(RC, 25-years-old, FG2)

### Ignorance of reproductive health care

Ignorance in maternal health issues contributed to lack of interest in antenatal follow up. Male partners felt compelled to attend the first visit because of HIV testing. Even though they were obligated to attend couple counselling and testing for HIV, a positive HIV test result raised fear and a risk of disharmony between couples, and subsequent deterrence to antenatal clinic attendance. One said:

“*Over seventy percent of men turn away not to be involved due to lack of knowledge of women health care*. *Men don’t have enough knowledge regarding women health*”

(RF, 23-year-old, FG1).

Another said:

“*For example*, *someone wants you to go for first clinic visit and take a test (HIV test)*, *a lot of young man fear testing issues (Couple testing for HIV)*. *Thus they stay away from partner*.”

*(*RE, 25-years-old, FG2)

### Factors out of their control

Male partners expressed being unaccepted and misperceived when playing their supportive role in antenatal care. They hopelessly felt as outsiders and temporary antenatal clinic visitors for exclusively testing for HIV. These external inhibitive factors eliminated their desire to be involved in antenatal care. One vented:

“*When people see me carrying a baby to the clinic they ask me*, *‘where is the baby’s mother*?*’ When I say that his mother has gone to work*, *people don’t not understand*.*… Going back to antenatal clinic issue*, *it seems like an activity for females and a man is not needed*, *unless it’s the first visit when couple test for HIV*”

(RA, 40-old, FG2).

Another said:

“*The challenges that we men get if we go to the clinics include finding women saying*, *‘This one pretends to love so much’*. *Then you find yourself not going there every time*, *because of these words*. *You see yourself disappearing*., *You see me the first time*, *second time and next time I say*, *‘Go alone’*”.

(RE 33years old, FG2)

Self-employed partners prioritized a day’s work to antenatal attendance in an attempt to fulfil their perceived societal role as bread earners. Skipping work implied lost earnings. Furthermore, male partners who were employed required permission from their employer to attend antenatal clinic. Respondents express frustration and pressure to choose employer’s demands over supporting their partners during antenatal care. One said:

“*I would have liked to go to the clinic every day when my wife goes*. *But If I go*, *what is the state in my pocket*. *Others are employed*. *But If I am self-employed*, *then if I do not work*, *I have nothing; and so she has to go alone*. *It is not that we purposefully ignore the clinic*.”

(RS 26-years-old, FG2).

Another added:

“(…) *Priority is to support wives*, *when during pregnancy*, *however*, *when I ask to go with my wife to the clinic*, *the other side (employers) says*, *‘Stay at work’*. *Who do you think will go*? *They use so much energy to educate us but it is not recognized at work*.*’*”

(RF, 35-years-old, FG2)

### Couple interaction and conflicts

Male partners had mixed opinions of how to interact and deal with emotional stress and mood changes that were claimed to be a source of tension and couple conflict during pregnancy. Some respondents explained how emotional stress limited male involvement in physical support. Others reported difficulties in adhering to antenatal care, when couples do not live together. One explained:

“*Let me say this*, *for other women*, *when they get pregnant you find like*, *a lot of anger and hate and some things they exaggerate……*. *it is a problem because this person has carried your baby*. *So you have to keep holding on but sometimes it becomes impossible*.”

(RA, 35- years-old, FG2).

Another added:

*I want to talk about challenges faced by young man*, *when you find they met a young women and have a sexual relation while she lives in … (mentioned an area south of Dar es Salaam) while you leave in… (mentioned an area north of Dar es salaam)*, *and you cannot live together*. *So it is difficult to do*, *for example*: *‘This and this exercise’ when you live separately*. *So first problem is distance*. *Since you do not live as couple*, *interaction will not go well*.

(RB, 35-years-old, FG 2)

On the other hand, other participants insisted on unwavering physical and emotional support despite tension in their relationship. Further, physical and psychological stress in pregnancy was perceived as an opportunity for physical and emotional support to their spouse. One explained:

“*That may be true*, *but each woman should be handled differently*. *I have a different outlook of this*, *Most of the Africans do not want to carrying their burdens*, *because of taking things for granted thinking that*, *if I am here or not here things will move*, *anyway*.”

(FK, 50- years-old, FG 1).

Another added:

“*There are others who believe that if they give out money their task is finished*. *(…) that is not enough*, *a pregnant woman needs more support from the partner*”.

(RG, 36-years-old, FG2)

### Institutional obstacles

Some respondents complained of having to scramble for antenatal service because of the overwhelming number of clients leading to queuing and a long wait time. They expressed challenges for men to sacrifice income-generating activities for a whole day, if they were to fully participate in antenatal clinic. They also reported partners sometimes discouraging their attendance at antenatal services because it would mean losing a day’s earnings, and might mean disrespect from care providers. Staff attitude was another deterrence from antenatal care. Respondents complained of bad language and disrespectful treatment, not necessarily at MNH but elsewhere, either during the recent or previous pregnancies. One said:

“*If services are improved and people come for a short time and will be treated and go*, *people will come (at ANC)*. *Even if a man is given priority to be served when they come with their wives*, *what if all women come with their men*? *It will mean that we do queuing for ages*.”

(RD, 37-years old, FG1)

Others shared their experience:

“*One thing is the bad language of those who serve us*, *the nurses*.”

(RD, 35-years-old, FG1)

“*So the service provider’s behavior contributes a lot and it is a reason for our partners not letting us accompanying them*.”

(RA, 40-year-old, FG2)

## Discussion

### Prevalence and determinants of male involvement

There was a high prevalence (69%) of male involvement in antenatal care for male partners whose women delivered at a tertiary health facility in city of Dar es Salaam. Concurring with these findings, similar rates were reported in urban and peri-urban parts of east and west Africa including Uganda, (65%) and Nigeria (64%) [33, 34]. This study was conducted in urban settings with higher likelihood of male partners’ awareness of importance of their involvement in the antenatal care, contrary to situations in peri- urban [35] and rural Tanzania [36], Ghana [8], Ethiopia [12] where prevalence of male involvement was between 18–45%. The observed variation could be explained by the methodological and cultural differences. Further, majority of women in this study were of young age (26–30 years old), married and with a college/university education, hence more likely to discuss, negotiate and involve their partners in deciding on maternal health issues and health seeking behavior [5]. Given that the majority of studied women had a good antenatal attendance (equal or more than four visits), it was likely to find male partners were encouraged to do the same. In this study, all women were in some form of relationship with their partners, either physically or socially, which could explain a similarly high rate of joint decision-making despite comparatively less male involvement in physical, emotional and financial aspects during pregnancy. Coherence of women’s reports and male partners’ opinions was evident with regards to women’s antenatal experience and male partners’ perception and experience of during antenatal care on the subject of cultural perspective of antenatal clinic as women’s affairs, male partners’ ignorance of reproductive health issues and uncontrollable external factors including pressure of work.

### Male partners’ perspective of their involvement in antenatal care

In qualitative interviews, respondents’ perspectives of their involvement during antenatal care was articulated into four themes including cultural norms and gender roles, ignorance of reproductive health care, factors outside their control, couple interaction and conflicts and health institutional obstacles. Objectively, male involvement could be discussed using male involvement framework [24] in three categories: practical, physical and emotional involvement, couple communication and joint decision-making and cultural and institutional barriers.

#### Practical, physical and emotional barriers

Even though only three quarters (72%) of women reported being accompanied by their partner to antenatal clinic at least once, all of them had some form of relationship (physical and social) with their male partner, and the majority acknowledged financial support to cover logistical expenses and health care services costs. Despite limitations in practical and physical support from restrictive employment policies and physical separation of couple, male respondents appreciated the advantages of physical and practical support, including learning opportunities with regards to care of pregnancy and their role to safe motherhood. Other African studies of similar cultural values and gender roles [37, 38] confirmed our findings. High rates of male physical involvement in antenatal care were reported in other peri-urban area of Myanmar [8], Rwanda [39] and Salvadori fathers’ [38], where physical attendance of male partners was considered an important factor in reducing maternal morbidity and mortality [39]. In this study, even though both women and male partners disapproved of outdated traditional masculinity, femininity in antenatal service was perceived to be an obstacle to physical male involvement as it has also been reported in other African settings [38, 40, 41].

Male partners expressed frustration with regards to demands of work that forced them to safeguard their daily earnings instead of attending antenatal visits with their partners. Thus, a hindrance to adequate practical, physical and emotion support that was also addressed by 17% of women whose partners were too busy to participate in antenatal care visits. Therefore, antenatal care processes and physical environment should encourage male presence and harmonize with male partners’ activities and societal role, in order to continue improving and sustaining high male attendance during antenatal clinic. Importantly, male partner inclusion within traditional women’s domains [42], should not compromise women’s right of reproductive choices.

#### Couple communication and joint decision making

In this study, nearly three quarters (70%) of women reported adequate communication with their partners. On the other hand, nearly half of them and some men acknowledged lack of male partners’ awareness of health care activities during antenatal visits. This could imply inadequate couple communication on issues related to antenatal care and subsequent lack of clarity of male partners’ roles. This would be a setback in joint decision making in antenatal care. Lack of clarity of male partners’ role and defined responsibility in pregnancy care has been reported to reduce their involvement in antenatal care in Myanmar [43] and Ethiopia [44]. It is essential to define the role of men in maternal health in these ways: a) addressing their expectations in the course of pregnancy and during childbirth, b) knowing when and where to seek care, c) being financially prepared to support for their partners, and d) giving feedback of quality of service provided to their partners [44].

Some men declared unbearable conflicts with their pregnant partners; others pledged unwavering emotional support, open communication and joint decision making with or without unforeseen stress related to pregnancy. Despite confirmation of support from some male partners, others perceived themselves as ‘lonely key players’ when interacting with both women and care providers. Thus, it is necessary to emphasize the care provider role in motivating male partners’ involvement during the process of care. Additionally, in low resource settings, joint decision-making can be dictated by financial capability [45] of a man or a woman; hence, a need for strategic and meaningful maternal health interventions that involve listening, defining, clarifying and reflecting on the perceived needs of women and men when setting and implementing maternal and newborn health services [46].

#### Social cultural and institutional factors

Similar to other studies [47, 48], social stigma surrounding HIV testing and a ‘positive’ test for HIV discouraged men from attending antenatal clinic. Women’s explanations for lack of male participation, included male perception of antenatal clinic as ‘woman affair’. Together or independently, both factors contributed to societal practices that lack a sense of belonging for male participants in antenatal services. Subsequently, the lack of sense of belonging diminishes male partners’ uptake of their roles and sense of obligation [49] to attend antenatal clinic regardless of whether they perceived maternal health care as “feminine.” [36]. Without harmonized health care polices and health education regarding the importance of men’s involvement in maternal health care, efforts to push for gender equality and access to reproductive health services will have a limited impact.

Despite the reported high antenatal attendance (85%) of male partners, there is still an opportunity to increase male participation if mistreatment from care providers such as bad language, disrespectful attitude and perceived lack of agenda for men is addressed in the health system. The poor experience of care was a negative for men themselves; but also discouraged women from urging their partners to attend antenatal care. Lack of physical space, poor structure of care with shortage of staff, sparse routine service points and disorganized clients’ pathways, either together or independently, could have contributed to long waiting time, unnecessary delays and lost earnings. Thus, there is a need to improve standards of structure and processes of maternal health service [50], in order to acquire and sustain male partners’ desire to be involved in supporting their partners to make reproductive choices and fully utilize antenatal care services [51].

### Strengths and limitations

Combining women’s understanding with perspective of their male partners in antenatal care brought a true picture of situations affecting male involvement in a natural milieu of the relationships and interactions of couples with new babies. Using mixed methods supplied an opportunity for in-depth understanding and corroboration of one point of view to another during data analysis and inference [52]. Thus, mixed methods increased reliability and credibility of findings through triangulation of the different findings. Consolidated Criteria of Reporting Qualitative studies (COREQ) was used as guidance to ensure trustworthiness, relevance and transferability of the research methods and findings. Interviewing male partners of different ages and ranging relationship experiences during pregnancy and childbirth helped to diversify point of view by stimulating a credible discussion. Capturing the opinion of male partners as they participated in supporting their counterparts at a health facility. The use of Kiswahili increased conformity to a natural environment of maternal health service provision. Examining male involvement in antenatal care based on a global multidimensional male-involvement framework [24] increased objectivity our findings.

Despite the mentioned strengths, this study was conducted in health facility; hence a limitation for generalization to the population. Interviews were conducted in Kiswahili and then translated into English, which increased the likelihood of loss of some meanings and conversational distinctions during translation. Since all authors are fluent in Kiswahili, this limitation was minimized. The study captured men who were supporting their partners during antenatal clinic and after delivery; hence, the participants were generally motivated to be involved in antenatal and postnatal care. Therefore, there was a risk of biased perspective of barriers for antenatal care involvement that could have differed from non-participants. However, capturing involved male participants might have enriched our findings of the real experience and situations during antenatal services, compared to that of male partners who had limited attendance to antenatal and postnatal services. The study suggests that the findings were adequate in drawing a conclusion of main barriers of male involvement in antenatal care.

## Conclusion and recommendations

The prevalence of male partners’ involvement in antenatal care was relatively high. Male involvement depended on their access to antenatal care education, standards of structure and process of antenatal service and how well their role was defined in the maternal health care system. Interactions and practice in society, the employment sector and the government health system should complement strategies that define and promote men’s involvement in maternal health.

We recommend health promotion measures that empower men with meaningful information regarding expectations and appropriate planning for pregnancy and birth preparedness. Future quality improvement measures at facility levels or within the health system should enforce inclusion of relevant and appropriate male agenda in antenatal care and childbirth. Further, there should be a sound implementation and monitoring system that uses relevant models in reproductive health services, including but not limited to the WHO Three level delay model and standards of maternal and child care. We also recommend incorporation of locally relevant strategies to encourage men’s attendance in maternal and child health care through local community meetings that discourage unfavorable gender norms, and by providing community-based maternal health education and role modeling at various levels of social and political leadership. There is still a need for further exploration of cultural attributes and the social role of paternity in specified communities, in order to contextualize interventions to improve male involvement in maternity care in different family models and pre-defined roles within the health system.
